# Supplementary material for: Transcriptome Profiling Unveils Key Genes Regulating the Growth and Development of Yangzhou Goose Knob
Source: Int J Mol Sci. 2024 Apr 10;25(8):4166. doi: 10.3390/ijms25084166 (PMC11050116; doi:10.3390/ijms25084166)
Supplement: Supplementary file 1 [file ijms-25-04166-s001.zip › supplementary figures.pdf]

Figure S1. Density chart of mRNA expression level.

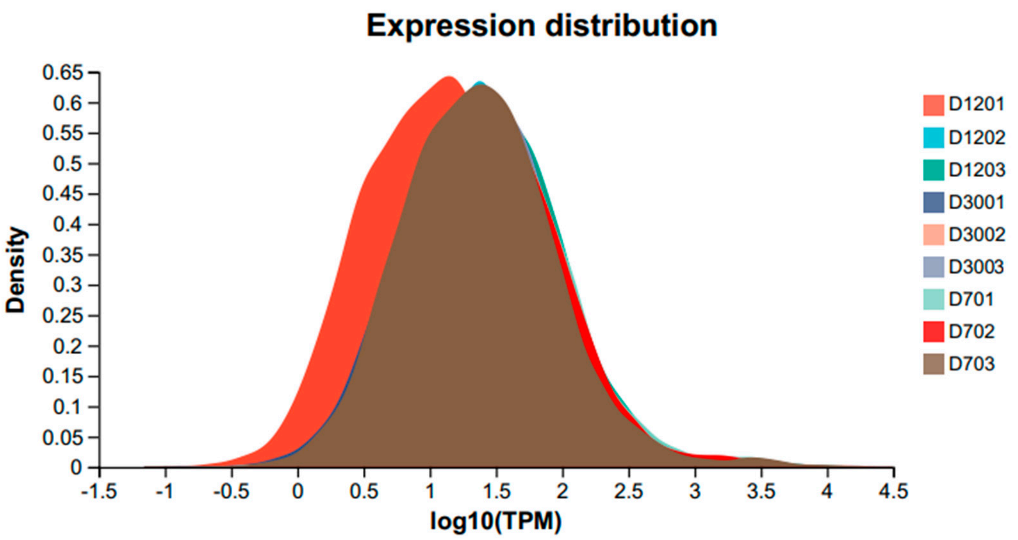

Figure S2. Box plot of mRNA expression level.

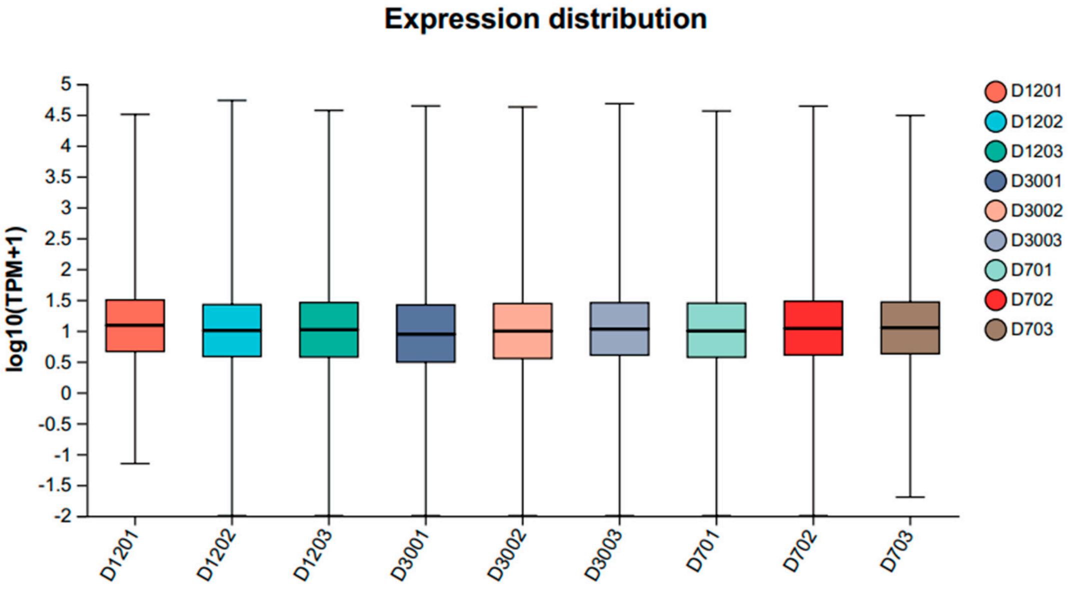

Figure S3. GO enrichment functional analysis of DEGs.

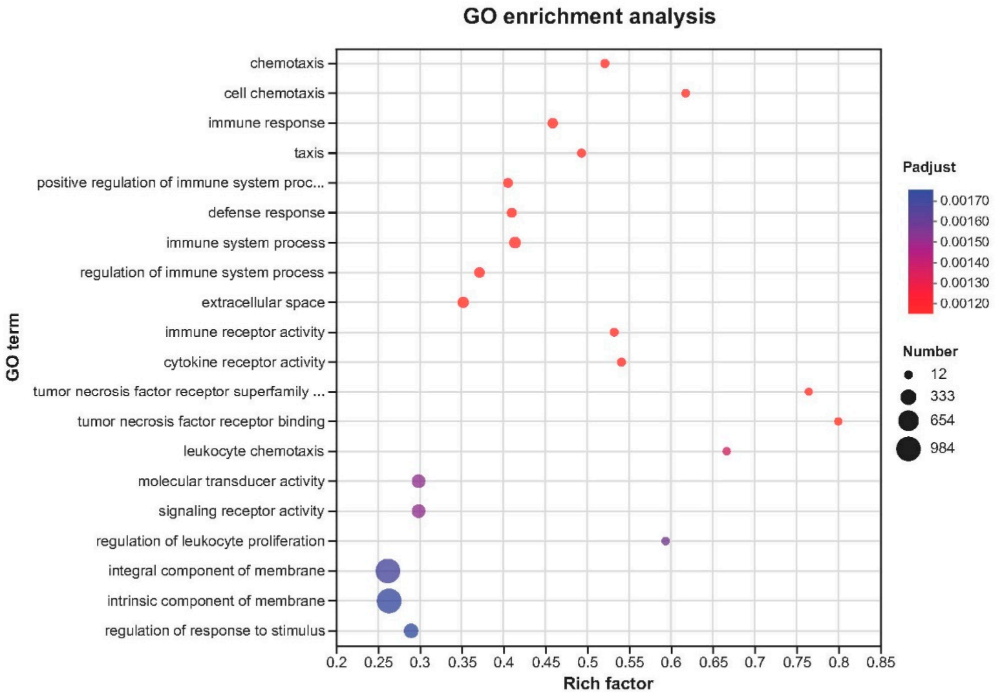

Figure S4. KEGG enrichment functional analysis of DEGs.

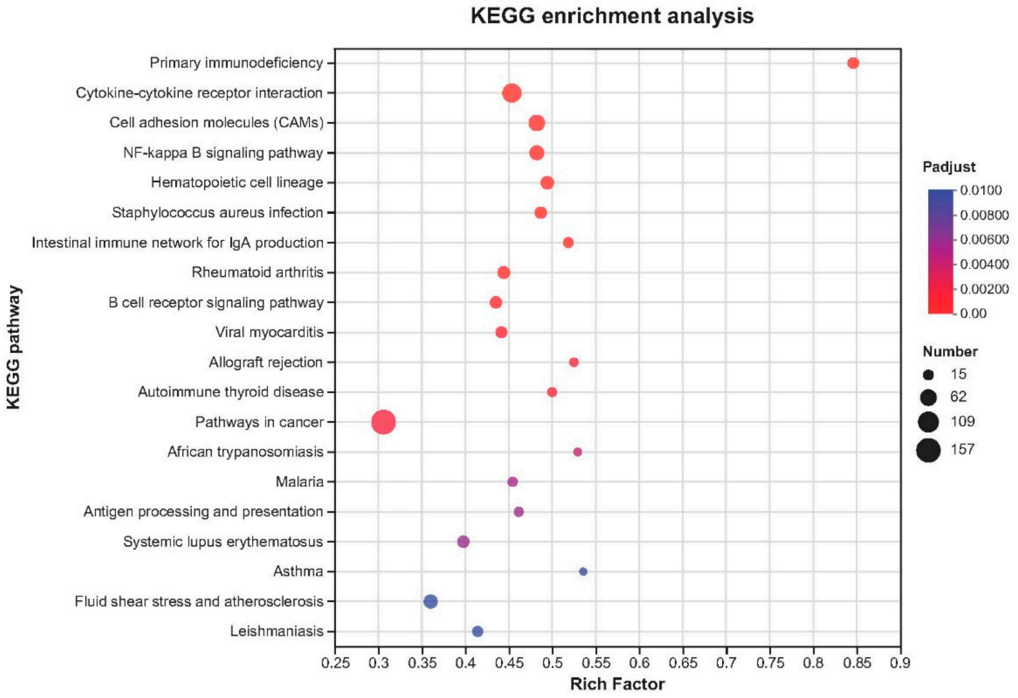

Figure S5. GO and KEGG network diagrams of each comparison group during the morphogenesis of goose knob. Note: Figure S5-a compares 70 vs 120, Figure S5-b compares

70 vs 300, and Figure S5-c compares 120 vs 300.

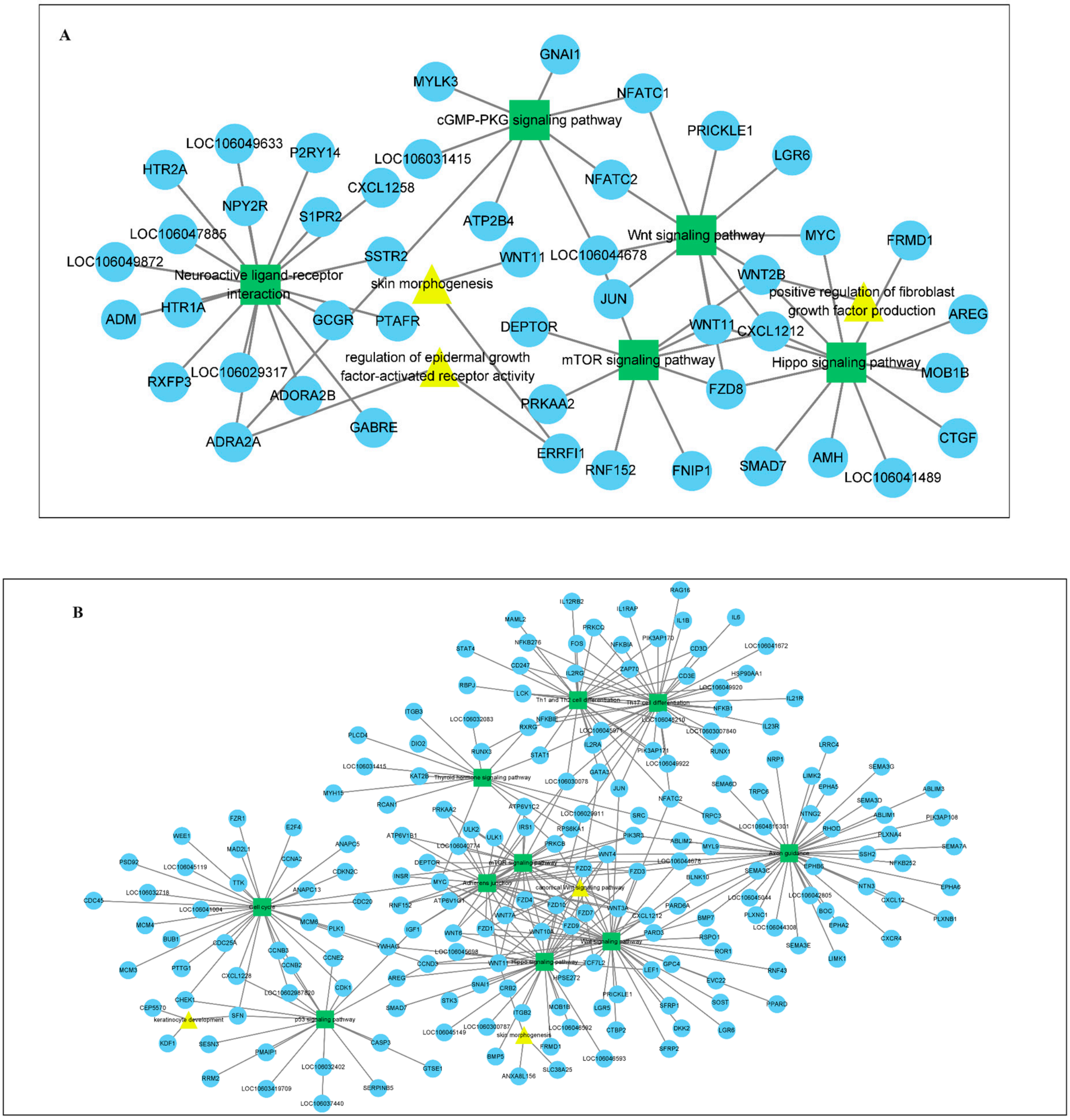

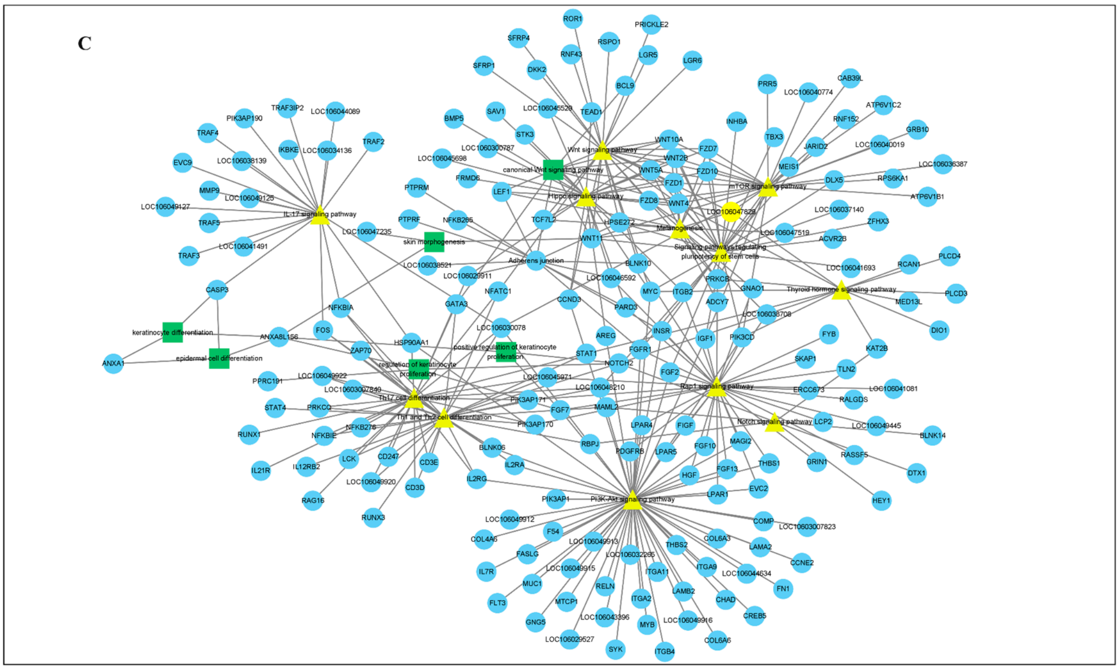

Figure S6. Time series expression analysis of differentially expressed mRNAs among three comparison groups.

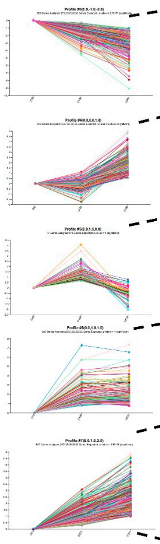

Enriched GO terms (P-Value)	Gene
regulation of cytokine secretion（2.65E-07）<br>regulation of cytokine production（1.97E-06）<br>regulation of response to stress（1.99E-06）<br>response to other organism（4.13E-06）	IL1R2、F2R、IL18、<br>TRAF3、IL1R2、IL18<br>PTN、WNT7A<br>TRAF3、IL1B
regulation of wound healing, spreading of epidermal cells(0.02)<br>negative regulation of wound healing, spreading of epidermal cells(0.02)	PHLDB2<br>PHLDB2
replication-born double-strand break repair via sister chromatid exchange（0.004）<br>cell cycle DNA replication maintenance of fidelity（0.004）<br>retinoic acid catabolic process（0.004）	RAD51<br>RAD51<br>CRABP1
neurofilament bundle assembly（0.0002）<br>cellular response to alcohol（0.0003）<br>regulation of catabolic process(0.0003）<br>response to forskolin（0.0005）	NEFL、NEFM<br>PRKAA2、ADCY5、GNAI1<br>PPP1R3B、PRKAA2<br>ADCY5、GNAI1
esophagus smooth muscle contraction（0.001）<br>choline metabolic process（0.001）<br>copper ion import（0.001）<br>pteridine-containing compound metabolic process（0.001）<br>small molecule catabolic process（0.001）	SULF2、SULF1<br>DMGDH、CHDH<br>ATP7B、STEAP2<br>MTHFR、MTR、PCBD1<br>GCAT、GLDC、HSD17B4
