# Supplementary figures and images for: Transcriptome Profiling Unveils Key Genes Regulating the Growth and Development of Yangzhou Goose Knob
Source: Int J Mol Sci. 2024 Apr 10;25(8):4166. doi: 10.3390/ijms25084166 (PMC11050116; doi:10.3390/ijms25084166)

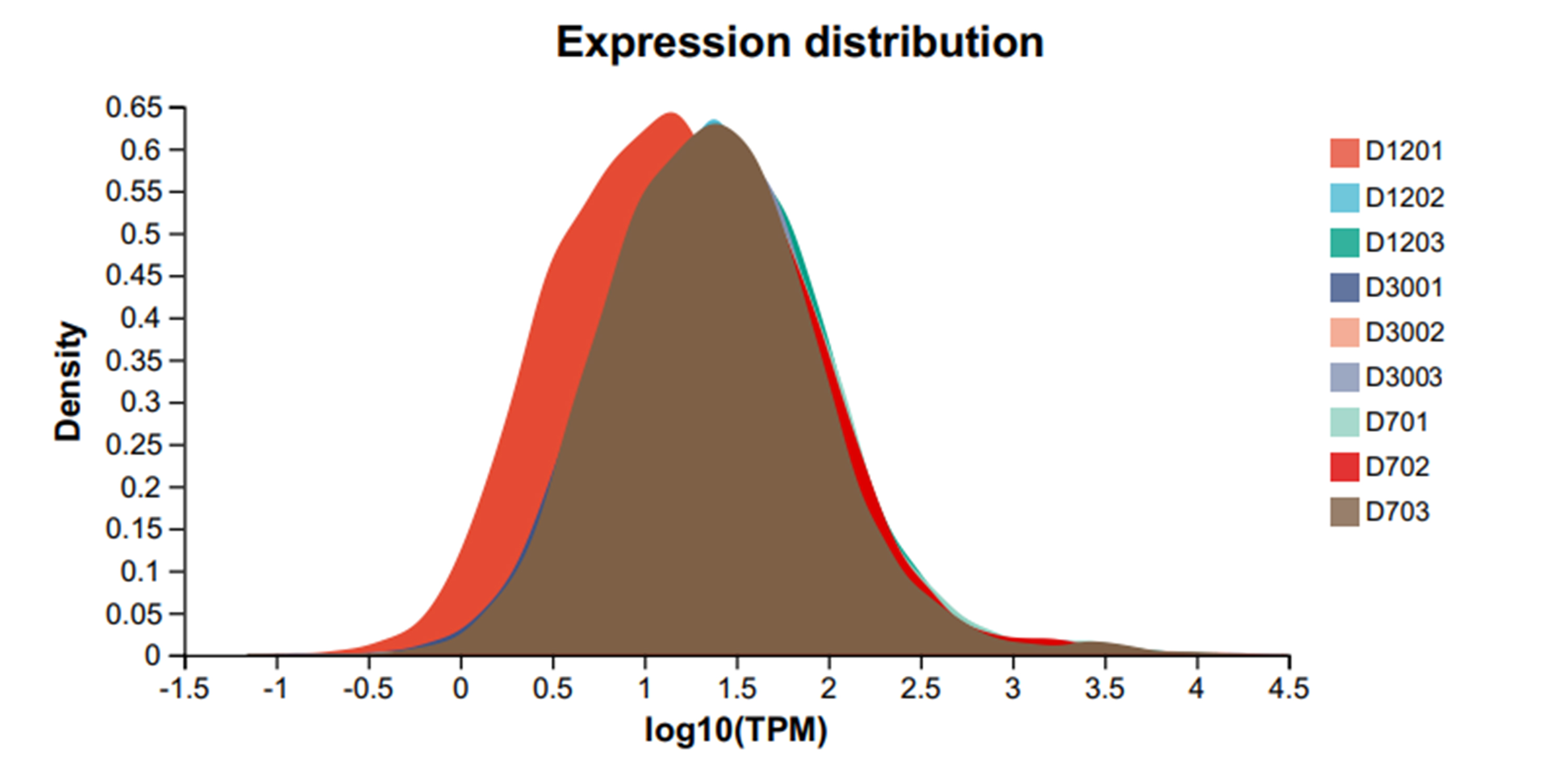

Supplement: Supplementary file 1 [file ijms-25-04166-s001.zip › Figure S1.jpg]

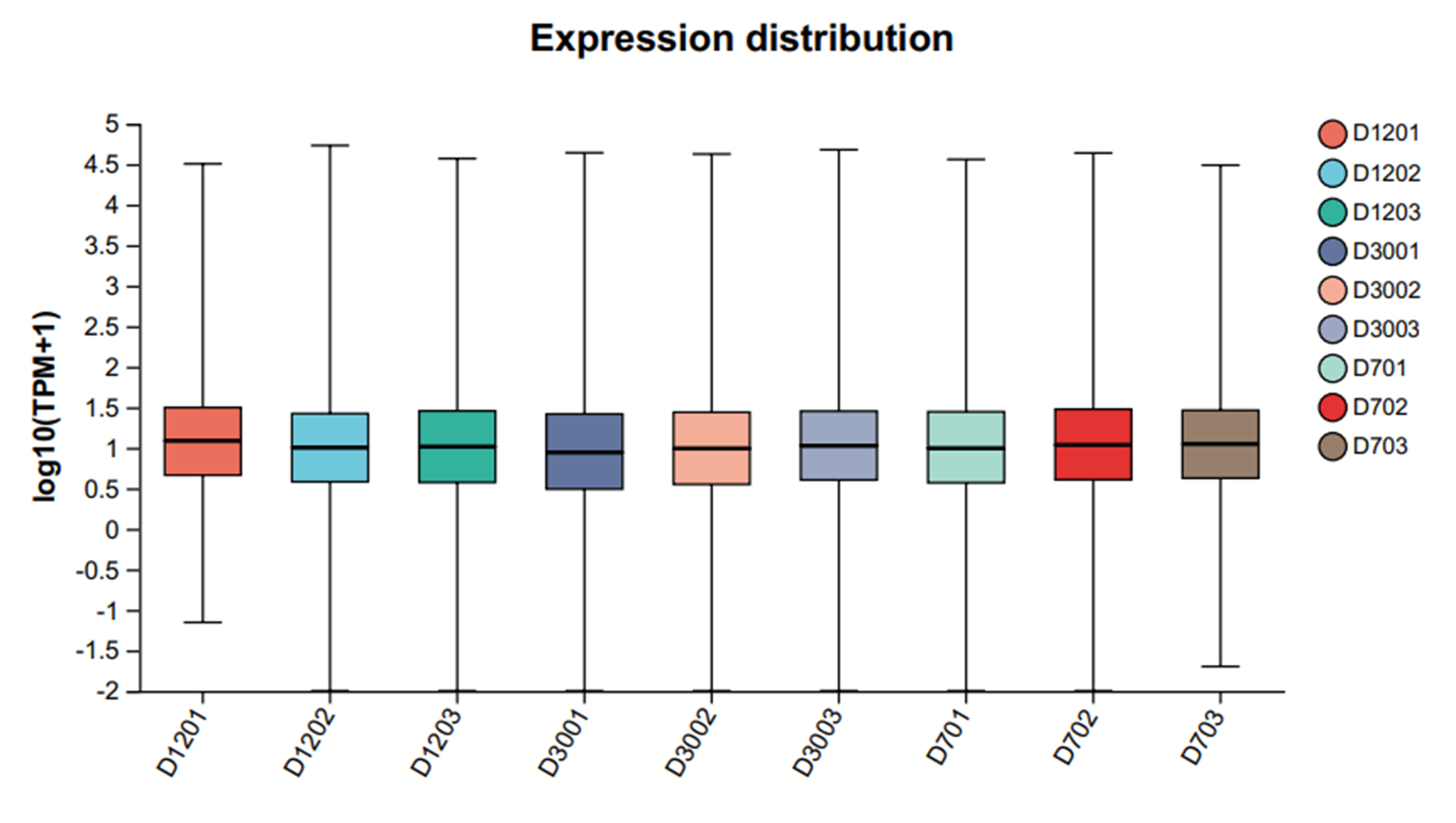

Supplement: Supplementary file 1 [file ijms-25-04166-s001.zip › Figure S2.jpg]

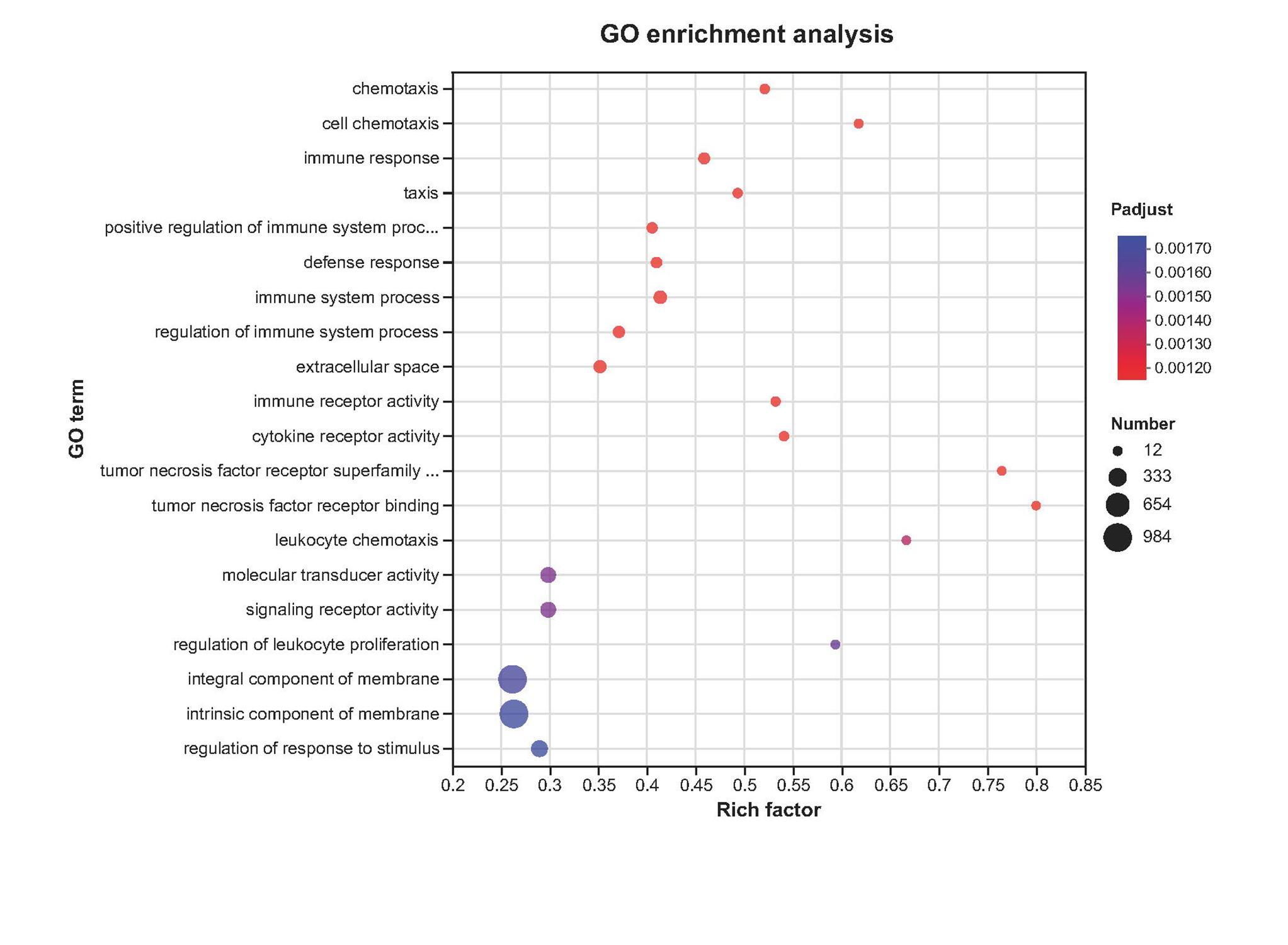

Supplement: Supplementary file 1 [file ijms-25-04166-s001.zip › Figure S3.jpg]

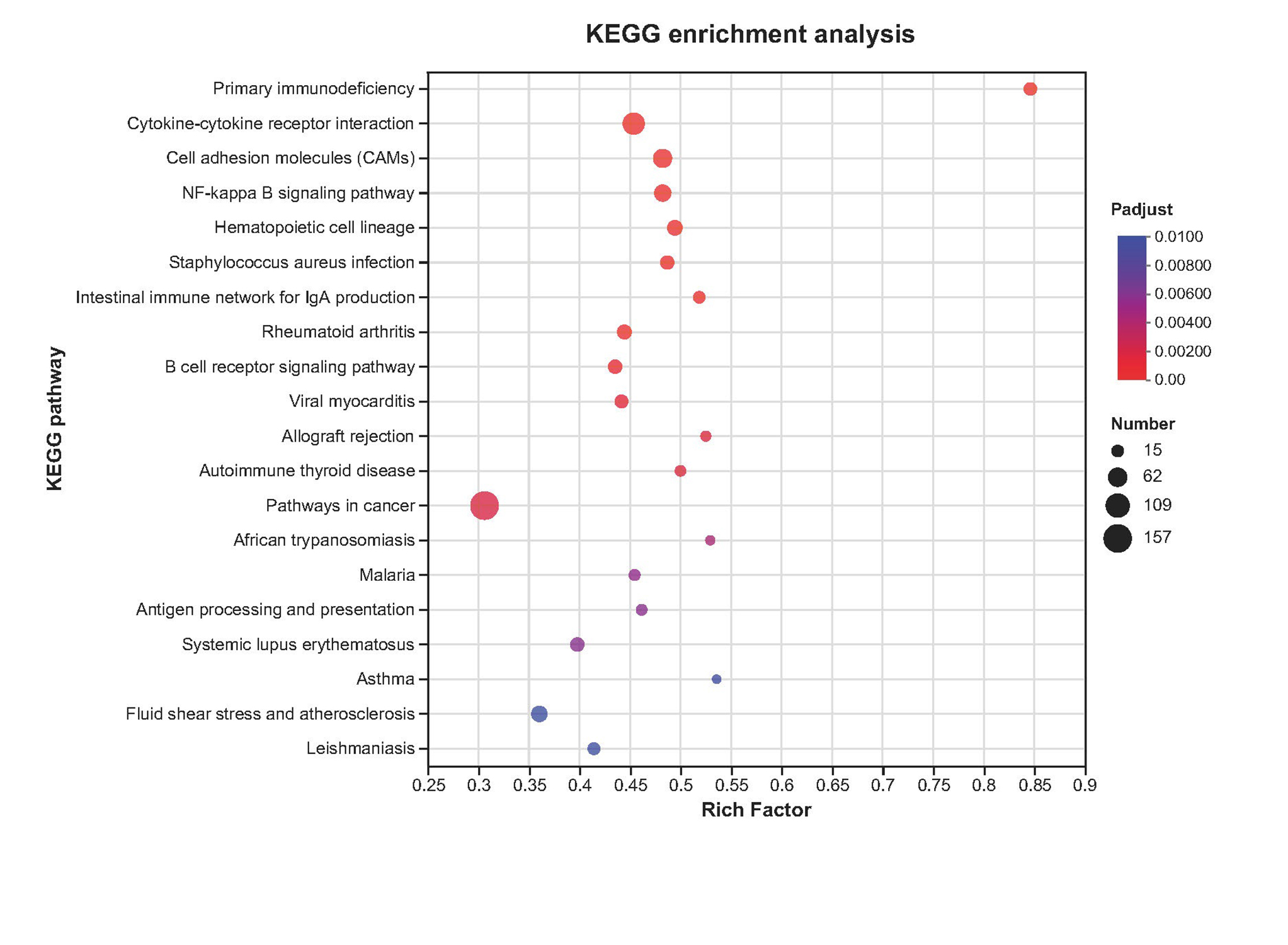

Supplement: Supplementary file 1 [file ijms-25-04166-s001.zip › Figure S4.jpg]

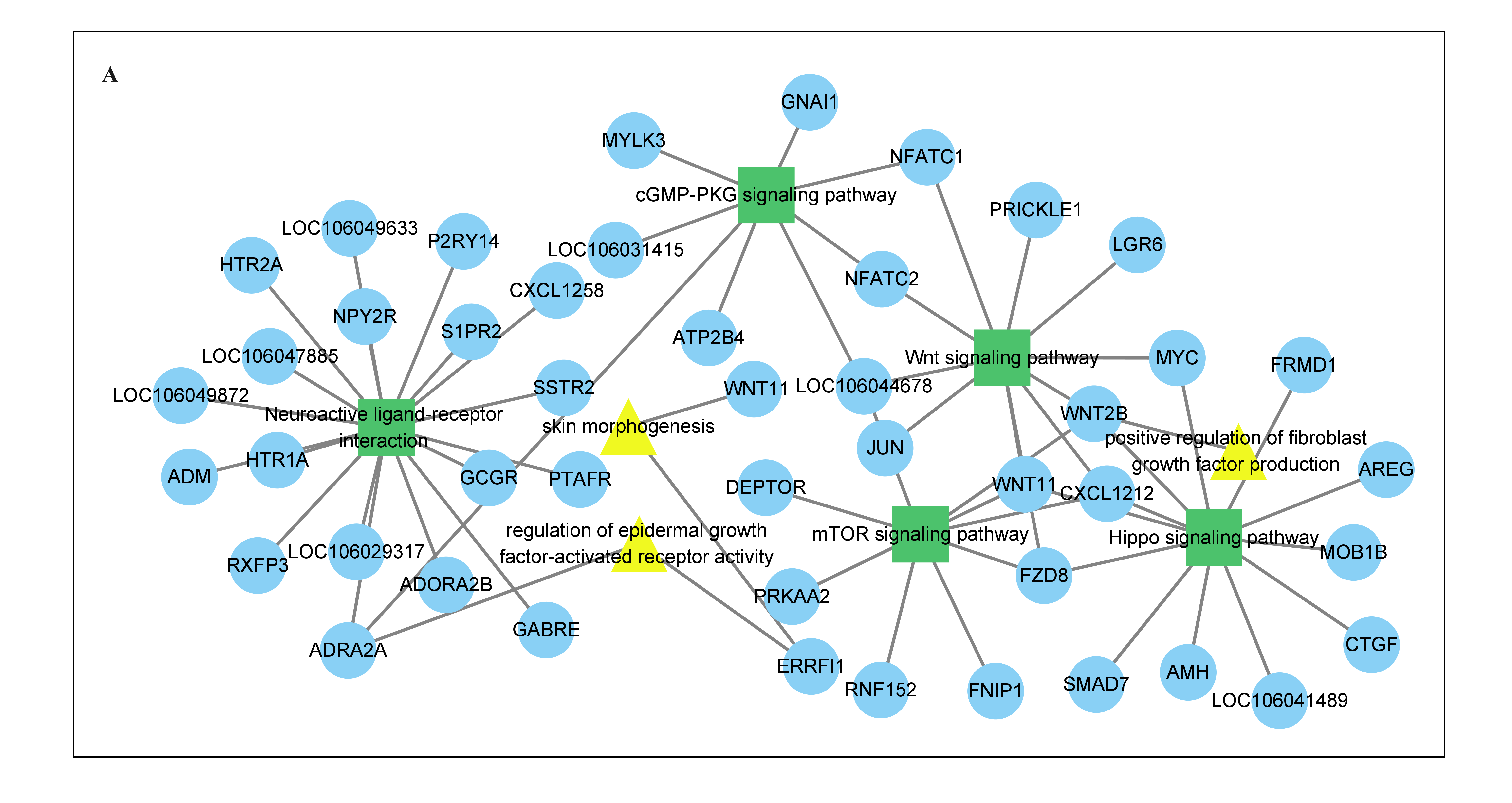

Supplement: Supplementary file 1 [file ijms-25-04166-s001.zip › Figure S5-A.png]

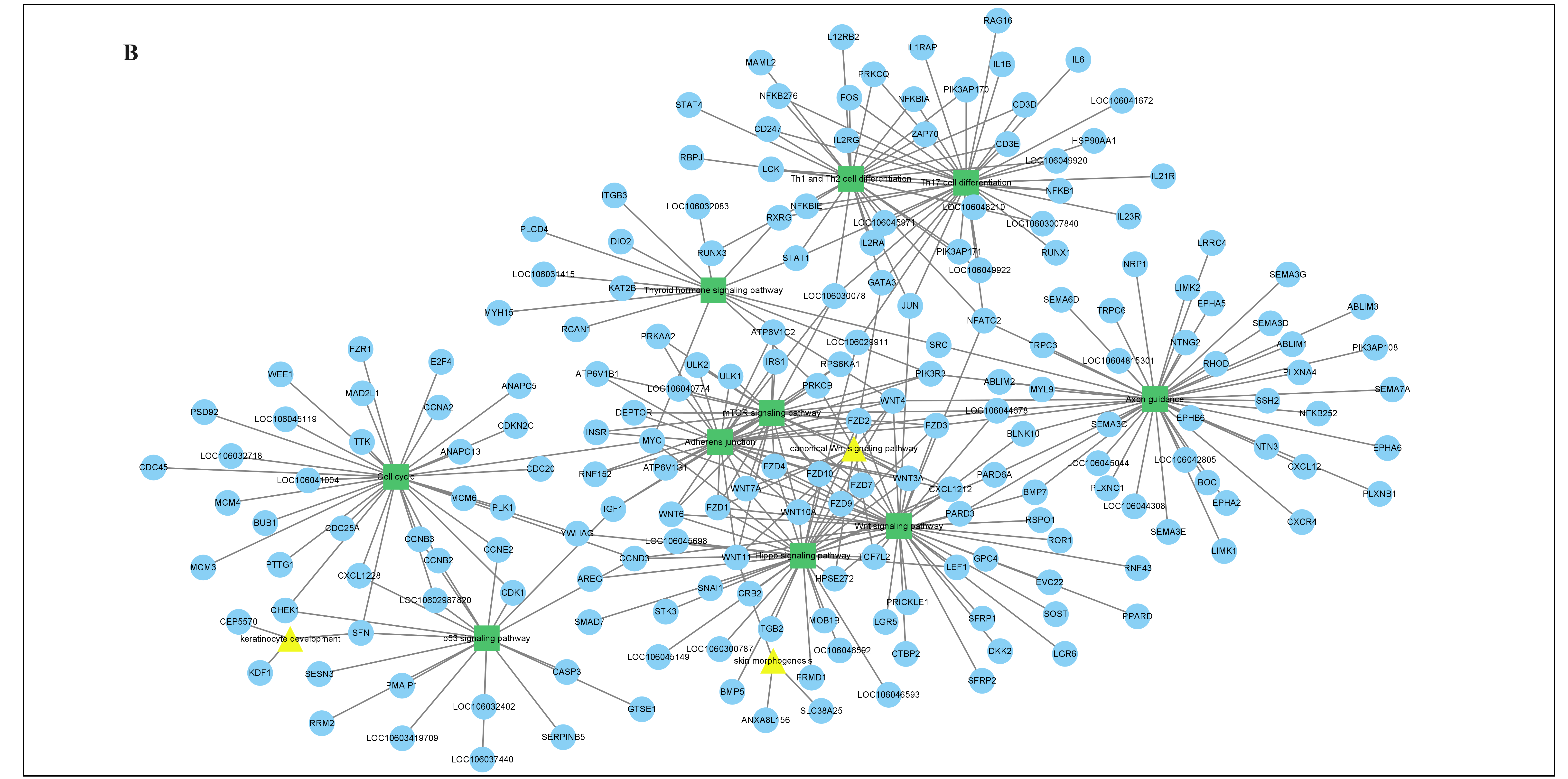

Supplement: Supplementary file 1 [file ijms-25-04166-s001.zip › Figure S5-B.png]

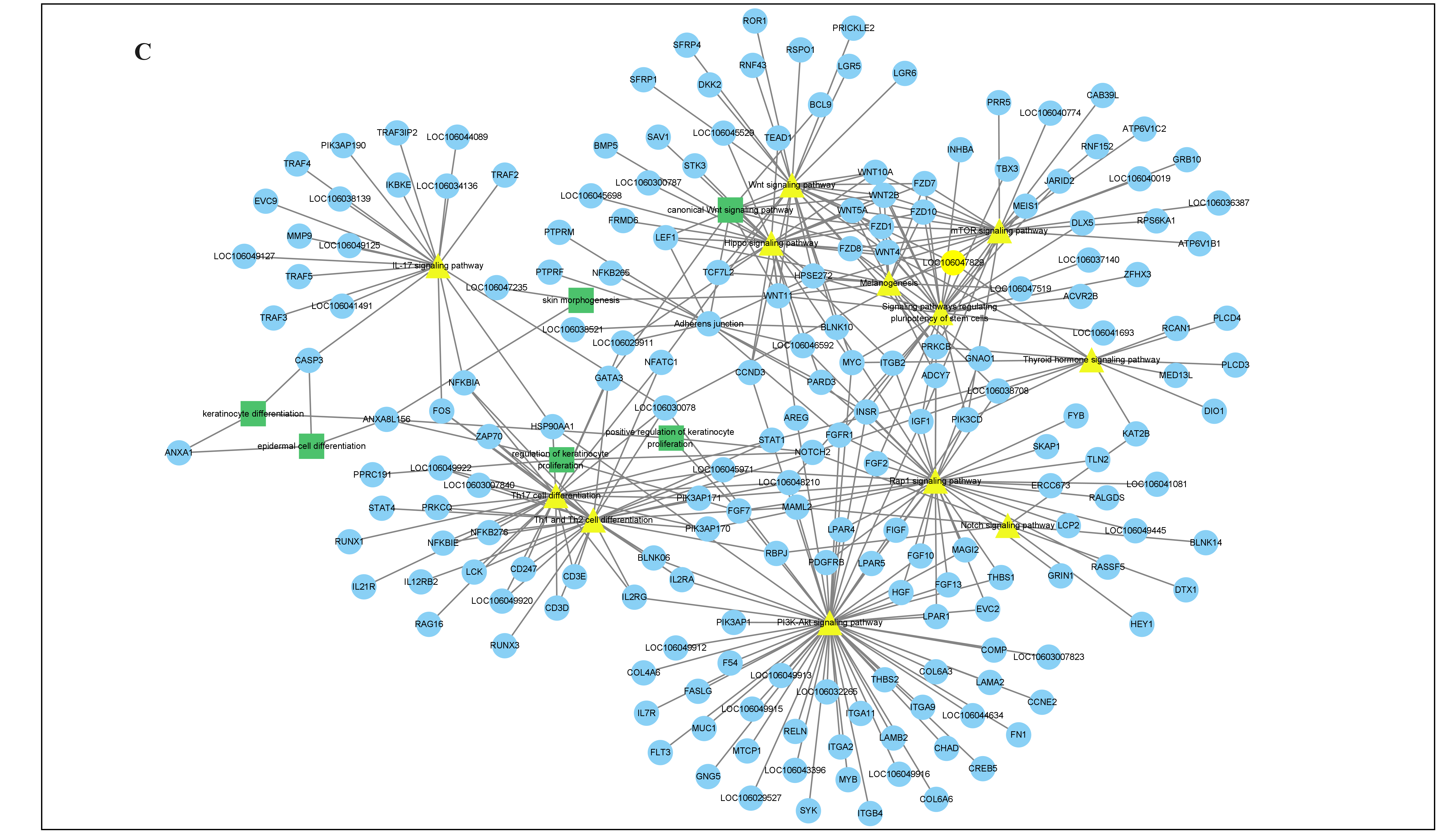

Supplement: Supplementary file 1 [file ijms-25-04166-s001.zip › Figure S5-C.png]

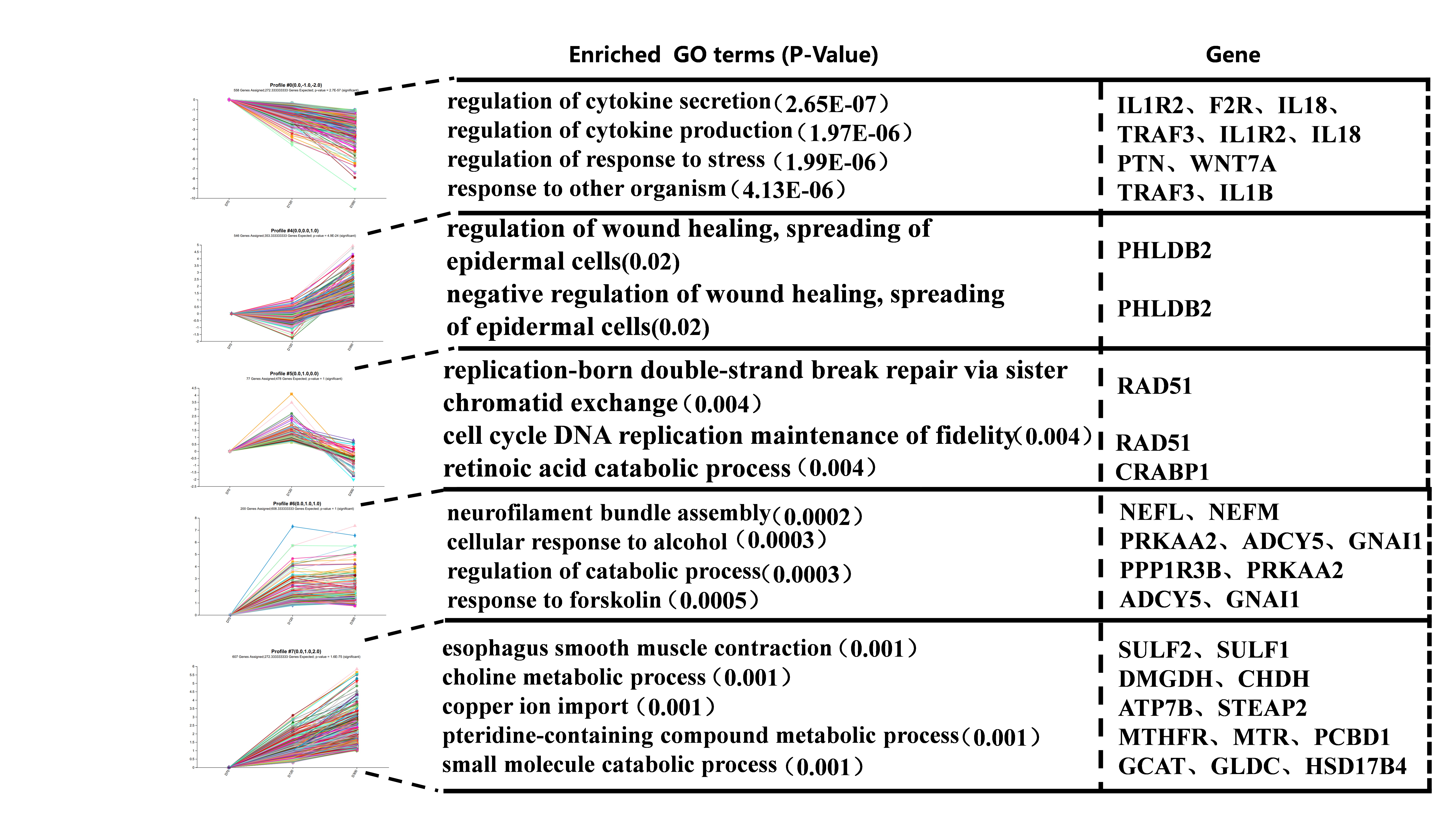

Supplement: Supplementary file 1 [file ijms-25-04166-s001.zip › Figure S6.jpg]
